# Croatian Society of Medical Biochemistry and Laboratory Medicine: National recommendations for blood collection, processing, performance and reporting of results for coagulation screening assays prothrombin time, activated partial thromboplastin time, thrombin time, fibrinogen and D-dimer

**DOI:** 10.11613/BM.2019.020503

**Published:** 2019-06-15

**Authors:** Ana Bronić, Desiree Coen Herak, Sandra Margetić, Marija Milić

**Affiliations:** 1 Department of Clinical Chemistry, Sestre milosrdnice University Hospital Center, Zagreb, Croatia; 2 Department of Laboratory Diagnostics, University Hospital Centre Zagreb, Zagreb, Croatia; 3 Department of Clinical Laboratory Diagnostics, Osijek University Hospital, Osijek, Croatia; 4 Faculty of Medicine, University of Osijek, Osijek, Croatia

**Keywords:** blood coagulation tests, haemostasis, standardization, harmonization, guidelines

## Abstract

A modern diagnostic laboratory offers wide spectrum of coagulation assays utilized in the diagnosis and management of patients with haemostatic disorders, preoperative screening and anticoagulation therapy monitoring. The recent survey conducted among Croatian medical biochemistry and transfusion laboratories showed the existence of different practice policies in particular phases of laboratory process during coagulation testing and highlighted areas that need improvement. Lack of assay standardization together with non-harmonized test results between different measurement methods, can potentially lead to incorrect decisions in patient’s treatment. Consequently, patient safety could be compromised. Therefore, recommended procedures related to preanalytical, analytical and postanalytical phases of prothrombin time, activated partial thromboplastin time, thrombin time, fibrinogen and D-dimer testing are provided in this review, aiming to help laboratories to generate accurate and reliable test results.

## Introduction

Haemostasis is the physiological response to an injury. It is a result of a complex interaction between vessels, platelets and coagulation plasma factors with its main function to stop bleeding on the site of vascular injury, while maintaining blood flow in intact blood vessels (*1*).

A modern diagnostic laboratory offers wide spectra of coagulation assays utilized in the diagnosis and management of patients with haemostatic disorders, preoperative screening and anticoagulation therapy monitoring (*2*). Among them, prothrombin time (PT), activated partial thromboplastin time (aPTT) and fibrinogen are the most commonly performed screening assays that could provide rapid, although non-specific information of the nature of haemostatic disorders (*3*). Results of these screening tests in conjunction with the patient’s medical history, will further direct the selection of more specific coagulation tests. A large number of distinct tests, often using a variety of methodologies, are commercially available (*4*). Lack of assay standardization together with non-harmonized test results between different measurement methods, can potentially lead to incorrect decision in patient’s treatment. Consequently, patient safety could be compromised, causing also additional healthcare costs (*5*).

The recent survey conducted among medical biochemistry and transfusion laboratories in Croatia showed the existence of different practice policies in particular areas of coagulation testing and highlighted areas that need improvement (*6*). As standardization and harmonization of the overall haemostasis testing process at national level should be implemented, recommended procedures related to preanalytical, analytical and postanalytical phases of coagulation screening assays PT, aPTT, thrombin time (TT), fibrinogen and D-dimer testing are presented in this review.

## Materials and methods

Recommendations on procedures in preanalytical, analytical and postanalytical phases of coagulation screening assays PT, aPTT, TT, fibrinogen, and D-dimer testing were created by the Working Group for Laboratory Coagulation (WGLC) formed by the Croatian Society of Medical Biochemistry and Laboratory Medicine (CSMBLM). Creation of the recommendations was prompted by the results obtained in the survey carried out by WGLC in 2015 among Croatian medical biochemistry and transfusion laboratories performing coagulation testing (*6*). Data provided in this review were collected by searching the PubMed and Ovid databases, as well as relevant publications of the Clinical & Laboratory Standards Institute (CLSI), British Society for Haematology (BSH) and International Society on Thrombosis and Haemostasis (ISTH). Key words used during the search were preanalytical, analytical and postanalytical phases of haemostasis or coagulation testing, coagulation assays, haemostasis assays, PT, international normalized ratio (INR), aPTT, TT, fibrinogen, D-dimer test, haemostasis or coagulation assays guidelines, interpretation of results in coagulation standardization and/or harmonization in haemostasis testing.

## Preanalytical phase in coagulation testing

The preanalytical phase of testing includes all processes from the time when a physician makes a request for certain laboratory test, until the time when sample is ready for testing. The majority of laboratory errors occur in this phase and problems can arise at any time prior the specimen is received in the laboratory (*5*, *7*). As most of errors occurring in preanalytical phase often become apparent later in the analytical and postanalytical phases, the preanalytical phase must have rigorous control mechanisms to avoid or reduce the risk of errors (*7*-*9*). Blood sampling is the most complex procedure in the preanalytical phase of testing and therefore it is most susceptible to errors. Standardized procedures for blood sampling are described in previously published National recommendations for venous blood sampling and should be followed (*10*). Only specific issues related to processes in preanalytical phase of coagulation testing will be discussed here.

### Test request

The recent survey conducted among diagnostic laboratories performing coagulation testing, showed that one of the main problem is related to the lack of information on the test request (*6*). Lack of appropriate information such as suspected diagnosis or anticoagulation therapy can lead to misinterpretation of test results as well as to unnecessary testing or retesting which could result in additional costs. Thus, additionally to specific coagulation tests requested, information on suspected or established diagnosis as well as on anticoagulant therapy use, should be an integral part of the coagulation test request (*10*). This is vital to ensure appropriate reporting and interpretation of test results.

**Recommendation**

Information on suspected or established diagnosis as well as on anticoagulant therapy use should be an integral part of the coagulation test request.

### Patient preparation and identification

General recommendations related to preparation of the patient prior to venipuncture and appropriate identification of patients during venipuncture are already described in National recommendations for venous blood sampling and are also applicable to coagulation testing (*10*).

**Recommendation**

Preparation of the patient prior to venipuncture and appropriate identification of patients during venipuncture should be in compliance with the National recommendations for venous blood sampling (*10*).

### Sample tubes and anticoagulant

Venous blood specimens for coagulation testing should be collected into glass or plastic tubes both containing non-activating surfaces. Such, glass tubes should be siliconized whereas plastic tubes should contain polypropylene as a non-activating material (*11*, *13*).

Test tubes should contain buffered trisodium citrate as anticoagulant, preferably at concentration of 105-109 mmol/L, also referred as 3.2% trisodium citrate. Although 129 mmol/L or 3.8% trisodium citrate tubes are also commercially available, reference intervals and test results may vary between samples collected in tubes with different citrate concentrations. For example, in samples collected into 3.8% sodium citrate tubes, PT and aPTT could be overestimated and fibrinogen could be underestimated if the reference intervals used is established with samples collected into 3.2% citrated plasma samples (*11*-*13*). Therefore, the major recommendation is that laboratories should standardize to always use test tubes with the same sodium citrate concentration, preferably 3.2% (*11*). This is important as only 3.2% trisodium citrate is used for thromboplastin international sensitivity index (ISI) assignment that is also recommended by Scientific and Standardization Committee (SSC) of the ISTH, and the CLSI have recommended the use of 3.2% trisodium citrate tubes (*11*).

The proportion of blood to anticoagulant volume in the test tube should always be 9:1. Exact amount of blood, usually indicated on the test tube must be drawn to ensure appropriate blood to anticoagulant ratio necessary for obtaining accurate test results (*11*). Although results of some recent investigations suggested that greater deviation from the fill line could be acceptable for particular tests, still the general recommendation is that +/- 10% of the fill line, corresponding to 90-110% fill volume could be acceptable (*12*-*14*).

**Recommendations**

1. Venous blood specimens for coagulation testing should be collected into glass or plastic tubes both containing non-activating surfaces.

2. Test tubes should contain buffered trisodium citrate as anticoagulant, preferably at concentration of 105-109 mmol/L, also referred as 3.2% trisodium citrate.

3. The proportion of blood to anticoagulant volume in the test tube should always be 9:1.

4. The deviation of +/- 10% the test tube fill line could be acceptable for the analysis.

### Blood sample collection

Blood samples should be obtained without trauma from a peripheral vein and away from an intravenous catheter if present. All relevant procedures related to venipuncture, including duration of tourniquet, and mixing of test tubes should be in compliance with previously published recommendations (*8*, *10*, *11*). For patients in whom a venipuncture site is not available, blood specimens have to be obtained through a vascular access device (central or peripheral venous catheter). If catheter was previously flushed with heparin further procedure should be followed. It is recommended to flush the central catheter with saline and to discard the first 5 mL of blood or the catheter dead space volume corresponding to 6-times of the line volume prior to coagulation tube collection (*11*, *15*).

When samples are collected from a capped off peripheral venous catheter, twice of the catheter and extension set dead space volume should be discarded. However, if blood must be drawn through the vascular access device, information should be always stated on the test request form and on the test report. Possible contamination with heparin or specimen dilution should be considered during result interpretation (*11*).

**Recommendations**

1. Collection of blood samples for coagulation testing should be in compliance to National recommendations for venous blood sampling (*10*).

2. Prior to collection of specimens for coagulation testing through an intravenous catheter, it is recommended to flush the central catheter line with saline and to discard the first 5 mL of blood or 6-times of the line volume. If samples are collected from a capped off peripheral venous catheter, twice of the catheter and extension set dead space volume should be discarded.

### Order of test tubes draw

In order to minimize contamination of specimens for coagulation testing, blood collection tubes should be filled in a specific sequence thus preventing erroneous test results due to carryover of additives between collection tubes and possible formation of micro-clots in the tubes (*10*-*14*). The coagulation tube has to be collected preferably before any other tube with additive (clot activators, *i.e.* thrombin) or anticoagulant agents such as ethylenediaminetetraacetic acid (EDTA), lithium-heparin and glycolysis inhibitors (*11*, *13*, *14*).

According to recent findings there is no need to first draw a discard tube prior to collecting specimens for routine coagulation tests and D-dimers (*10*, *11*, *14*). The exception of this rule includes a procedure when a winged blood collection set should be used, as the air in the tubing leads to the underfilling of the test tube. A discard tube in this case should be a non-additive (*11*).

**Recommendations**

1. The coagulation tube has to be collected preferably before any other tube with additive (clot activator or anticoagulant).

2. There is no need to first draw a discard tube prior to collecting specimens for routine coagulation tests and D-dimers. The exception of this rule includes a procedure when a winged blood collection set should be used, as the air in the tubing leads to the underfilling of the test tube. A discard tube in this case should be a non-additive

### Specimen rejection

Each laboratory should have defined criteria for rejecting unsuitable specimens for coagulation testing. Samples that do not arrive in appropriate timeframe for analysis (see chapter: Sample storage until analysis), unlabelled or mislabelled specimens, clotted specimens, specimens collected in the test tube with a wrong anticoagulant or those with inadequate blood to anticoagulant ratio, grossly haemolysed specimens and specimens that were refrigerated before testing should be rejected (*8*, *11*, *16*).

**Recommendation**

Each laboratory should have defined criteria for rejecting unsuitable specimens for coagulation testing.

### Sample processing

Most haemostasis screening assays, PT, aPTT, TT, fibrinogen as well as D-dimers, should be determined in plasma samples prepared by centrifugation of a primary collection tube at ambient temperature (18-25 °C), at 1500xg for 15 minutes (*8*, *11*, *16*). Although higher speed and shorter duration of centrifugation may be used, it is important to note that use of high centrifugal forces may induce platelet activation and lyses of erythrocytes (*8*, *11*, *16*). To obtain appropriate sample, centrifuge with swing-out rotor should be used and use of centrifuge breaks should be avoided (*16*). If used, refrigerated centrifuges should be set to maintain ambient temperature, since lower temperatures can lead to platelet activation and adverse effects (*11*). For all plasma samples that should be frozen until analysis, double centrifugation prior to freezing is preferable to obtain platelet poor plasma (PPP) containing < 10 x10^9^/L platelets (*16*, *17*). Plasma aliquot to be stored should not be taken near the cells.

**Recommendations**

1. Plasma samples for determination of screening assays PT, aPTT, TT, fibrinogen as well as D-dimers, should be prepared by centrifugation of primary collection tube at ambient temperature (18-25 °C), at 1500xg for 15 minutes.

2. All plasma samples that should be frozen until analysis, should be double centrifugated prior to freezing to obtain platelet poor plasma (PPP), containing < 10 x109/L platelets.

### Samples with high haematocrit values

In samples with haematocrit (Hct) values above 0.55 L/L, the final citrate concentration in the tube should be adjusted in order to maintain the appropriate blood to anticoagulant ratio at 9:1. This is important since in samples with elevated Hct values, blood to anticoagulant ratio falls below 9:1, causing excess amount of citrate for the volume of plasma in the tube that consequently could affect test results. As the first step in the procedure, the volume of citrate that should remain in the test tube have to be calculated by applying the following equation (*11*, *18*):

C (mL) = 0.185 x [blood volume (mL)] x [1.0 – Hct (L/L)],

where C is the volume of citrate in mL that remains in the tube, 0.185 is a constant, the blood volume depends on the test tube used, and Hct is the patient’s haematocrit (L/L).

The volume of citrate in mL that remains in the tube should be deducted from the total volume of citrate in the test tube, resulting in the citrate volume that should be removed from the test tube. It is recommended to use a tuberculin syringe for drawing out the anticoagulant, in order to maintain the vacuum in the collection tube. If tuberculin syringe is not available, automatic pipettes could be used for citrate extracting. Since the use of automatic pipettes for citrate adjustment means losing vacuum in the collection tube, blood should be drawn using an open system. After removing the tube cap and adjustment of the citrate dose, blood should be added to the tube mark. The tube should be manually recapped and sample thoroughly mixed. Further sample handling should be performed as with all other coagulation samples.

Regardless of how citrate extracting would be performed, the responsibility of the laboratory personnel is to inform users about the need of the correction of the citrate volume and appropriate procedure for further sampling. On the test report, it should be always indicated that citrate volume in the test tube was adjusted due to high haematocrit values: “Coagulation assays are performed in samples with adjusted volume of anticoagulant (citrate) due to high value of haematocrit (Hct = xx L/L).”, where xx represents the exact amount of haematocrit. If it is not possible to obtain a new specimen for correction of the citrate volume, appropriate comment should be reported along with the test results: “It is not possible to adjust citrate volume due to high haematocrit value (Hct = xx L/L). Inappropriate blood to anticoagulant ratio could have influence on test results. It is recommended to repeat sampling with prior agreement to laboratory personnel”.

**Recommendations**

1. The final concentration of the citrate in the test tube should be adjusted in order to maintain the appropriate blood to anticoagulant ratio at 9:1 in samples with haematocrit values above 0.55 L/L.

2. On the test report, it should be always indicated that citrate volume in the test tube was adjusted due to high haematocrit values: “Coagulation assays are performed in samples with adjusted volume of anticoagulant (citrate) due to high haematocrit value (Hct = xx L/L).”, where xx represents the exact amount of haematocrit.

### Haemolysis, hyperbilirubinemia and lipemia

Haemolysis, hyperbilirubinemia and lipemia can have impact on coagulation test results (*19*). Their presence could be detected by visual inspection or automatically. Analytical interferences mainly occur due to the spectral overlap of interfering substances (haemoglobin, bilirubin or lipid particles). The new generation of coagulation analysers measure optical absorbance at different wavelengths, as index of haemolysis, icteria and lipemia (HIL index), allowing the identification of these potentially problematic samples prior to analysis and the impact of interfering substances during testing will be reduced by auto selection of the appropriate wavelength (*19*, *20*). If there is no possibility for automated HIL determination, acceptable concentration of interfering substances should be determined by visual inspection. Beside spectrophotometric interference, haemolysed samples could be also problematic due to release of cellular components, resulting in premature coagulation activity and disruption of clot detection (*19*). Therefore, whenever this is suspected, a new not haemolysed sample should be drawn for testing (*11*). Apart to *in vitro* caused haemolysis, intravascular or *in vivo* haemolysis could be present as a result of certain medical conditions. The presence of intravascular haemolysis could be suspected and ruled out prior to resampling with appropriate communication and information from clinical personnel. In such cases, test results should be reported with appropriate notation on the test report (intravascular haemolysis).

The optical bias due to hyperbilirubinemia is mainly insignificant and could be prevented by measurement at alternative wavelengths while the test results may be reliably reported (*19*). Similar, the use of different wavelengths and/or higher sample dilutions in assays with plasma predilution (*e.g.* fibrinogen) could prevent optical bias due to lipemia (*19*). To remove the influence of lipemia, some clinical laboratories use ultracentrifugation, however data on the validation of this procedure have not been published (*16*).

However, use of mechanical and/or electromechanical clot detection methods is recommended whenever it is possible for samples that contain substances interfering with light transmission (*11*). Despite this possibility, biological interferences could still have influence on coagulation test results. For example, severe lipemia will affect coagulation by displacement of plasma which will be reflected in the prolonged clotting times in coagulation assays.

**Recommendations**

1. For coagulation screening assays laboratories should evaluate acceptable concentration of interfering substances for their own system (reagent/coagulometer).

2. If intravascular or in vivo haemolysis is suspected, along with the results appropriate notation on the test report (intravascular haemolysis) should be given.

3. Use of mechanical and/or electrome-chanical clot detection methods is rec-ommended whenever it is possible for samples that contain substances inter-fering with light transmission.

### Storage of specimens until analysis

In ideal conditions, testing of all coagulation assays should be performed within 4 hours of blood collection. According to recent investigations, the exception of this rule could be applied to PT and D-dimer testing, for which specimens can be stored at ambient temperature for up to 24 hours after blood collection, either uncentrifuged or centrifuged with plasma remaining on the top of the cellular component in an unopened tube (*11*, *12*, *20*). However, since sample stability may be dependent on measurement system in use (thromboplastin reagent and coagulometer), laboratories that accept specimens for PT and D-dimer testing stored within 24 hours of blood collection, should check sample stability with their own system (*21*).

Specimens for all haemostasis assays should be kept capped (in an unopened tube) at ambient temperature (18-25 °C) until analysis. Storage at refrigerated temperature (2-8 °C) is not recommended due to possible cold activation of coagulation factor VII (FVII) and also loss of coagulation factor VIII (FVIII) and consequent impact on PT and aPTT test results (*16*, *20*, *22*). If aPTT testing is requested for unfractionated heparin (UFH) monitoring, specimens should be centrifuged and plasma should be removed from cells within 1 hour of collection as there is possibility of heparin neutralization by platelet factor 4 (PF4) (*11*, *16*, *21*).

If testing for any haemostasis assay is not possible within the allowed time, plasma should be removed from the cells after centrifugation and immediately frozen at – 20 °C or below for short-term storage (up to two weeks) or at – 70 °C for up to six months. When multiple assays are requested or will be tested at different time, a separate aliquot for each test ordered should be frozen (*16*, *20*).

**Recommendations**

1. Specimens for all haemostasis assays should be kept capped (in an unopened tube) at ambient temperature (18-25 °C) until analysis.

2. Testing for all coagulation assays should be performed within 4 hours after blood collection. The exception of this rule could be applied to PT and D-dimer testing, for which specimens can be stored at ambient temperature for up to 24 hours after blood collection, either uncentrifuged or centrifuged with plasma remaining on the top of the cellular component in an unopened tube.

3. Laboratories that accept specimens for PT and D-dimer testing stored within 24 hours of blood collection, should check sample stability with their own system.

4. If aPTT testing is requested for unfractionated heparin (UFH) monitoring, specimens should be centrifuged and plasma should be removed from cells within 1 hour of collection.

5. If testing for any haemostasis assay is not possible within the allowed time, plasma should be removed from the cells after centrifugation and immediately frozen at – 20 °C or below for short-term storage (up to two weeks) or at – 70 °C for up to six months.

### Transport of specimens to distant laboratories

If coagulation specimens should be sent for analysis to distant laboratories, samples should arrive in the testing laboratory in real time to allow analysis within appropriate timeframes specified for each assay (*16*, *20*). If this is not possible, blood samples should be double centrifuged and plasma samples should be transferred into a polypropylene vial. Each vial should be labelled with the patient’s full name, date of birth, identification number (insurance number or unique identification number in laboratory). In addition, sample type (*e.g*. citrate plasma), date and time of sampling should be also provided. Plasma sample should be immediately frozen according to previously described procedure. Frozen plasma specimens should be transported preferable on a dry ice, and if not possible on sufficient amount of regular ice in a Styrofoam container in order to keep the samples solidly frozen until they arrive at the laboratory. Assays should not be performed on any specimen that does not arrive in coagulation laboratory solidly frozen (*5*, *8*, *11*).

**Recommendations**

1. If coagulation specimens should be sent for analysis at distant laboratories, samples should arrive in the testing laboratory in real time to allow analysis within appropriate timeframes specified for each assay. If it is not possible to transport specimens within appropriate timeframes for analysis, blood samples should be processed (frozen) according to procedure described in previous section.

2. Each vial with specimen that should be sent for analysis at distant laboratories should be labelled with the patient’s full name, date of birth, identification number (insurance number or unique identification number in laboratory). In addition, sample type (e.g. citrate plasma), date and time of sampling should be also provided.

3. Frozen plasma specimens should be transported preferable on a dry ice, and if not possible on sufficient amount of regular ice in a Styrofoam container in order to keep the samples solidly frozen until they arrive at the laboratory.

### Thawing of frozen plasma samples

Thawing of frozen plasma samples should be performed rapidly, for 10 minutes, at 37 °C in an incubator, dry thermo block or water bath (*5*, *16*, *20*). To ensure sample integrity prior to testing, thawed sample should be thoroughly and adequately mixed (*23*). Optimal procedure of mixing is the gentle inversion of sample for 180° and return to starting position for 6-times (*23*). However, if samples are not completely thawed or are left too long at 37 °C, spurious test results could be generated as this could lead to deterioration of coagulation factor activities and compromised sample integrity (*5*, *20*, *24*). If water bath is used for thawing, it is necessary to ensure the integrity of the patient data on the label.

**Recommendation**

1. Thawing of frozen plasma samples should be performed rapidly, for 10 minutes, at 37 °C in an incubator, dry thermo block or water bath.

2. To ensure sample integrity prior to testing, thawed sample should be thoroughly mixed by the gentle inversion of sample for 180° and return to starting position for 6-times.

## Analytical phase in coagulation testing

### Quality control

The purpose of quality control (QC) is to ensure reliable test results. Both internal (IQC) and external quality (EQC) control schemes should be fundamental parts of quality assurance in diagnostic laboratory performing coagulation assays. Internal quality control ensures continuous evaluation of the quality of the results and reduce random variation within or between days, whereas the main objective of EQA is to establish comparability of data between laboratories and accordingly, trueness of measurement (*27*, *28*). Internal quality control should be performed always following reagent opening or/and reconstitution, calibration, preventive instrument maintenance and repair (*27*). For quantitative tests, as a minimum two levels of control material including both normal and pathological ranges should be run every eight hours of continuous operation (*29*). However, different IQC schedule could be allowable if laboratory has analysed its own working process and performed the risk analysis prior to use. Considering EQA, there is still no evidence for an optimal EQA frequency, but it should be essential part of the total quality management system in laboratory (*2*).

**Recommendation**

1. Internal quality control (IQC) plasma should be analysed always following opening or/and reconstitution of new reagent, calibration, preventive coagulometer maintenance and repair.

2. For quantitative coagulation tests, as a minimum two levels of control material including both normal and pathological control plasma should be run every eight hours of continuous operation. Different IQC schedule could be allowable if la-boratory has analysed its own working process and performed the risk analysis prior to use.

3. External quality control should be es-sential part of the total quality manage-ment system in the coagulation labora-tory.

### Coagulation assays

In order to report accurate and reliable results of coagulation assays, it is pivotal for a laboratory to select an appropriate assay method. Various commercial assays that are available on market could be divided into three main categories: clotting, chromogenic and immune assays. Specific information related to test principles, equipment and techniques used in coagulation laboratories are in general described in Mackie *et al.* and are not part of current recommendations (*25*). For implementation of coagulometers into daily practice, evaluation and validation should be performed following previously published recommendations by Gardiner *et al.* (*26*).

When selecting a suitable method for coagulation assays it is important to know their analytical characteristics thus the most important analytical features of PT, aPTT, TT, fibrinogen and D-dimer assays will be listed below (*4*).

### Prothrombin time

The PT is the most commonly performed screening coagulation assay that is used for monitoring vitamin K antagonist (VKA) therapy and for assessing hereditary and acquired deficiencies of coagulation factors II (FII), V (FV), VII (FVII), X (FX) and fibrinogen or presence of their inhibitors (*29*). All commercial PT reagents (also known as thromboplastins), contain tissue factor, phospholipids and calcium chloride. Different commercial thromboplastin preparations derived from human, animal or recombinant sources are available. PT results are strongly dependent on the reagent type and coagulometer used and therefore will vary between laboratories (*4*).

In order to standardize PT and allow monitoring of VKA therapy across different laboratories the World Health Organization (WHO) has introduced the INR system for PT results reporting (*29*-*31*). For each PT reagent lot, manufacturers have to determine and assign specific ISI value, which indicates the sensitivity of the reagent of vitamin K dependent factor levels in comparison to the appropriate WHO reference standard, the International Reference Preparation (IRP). The INR is a calculated value, representing the prothrombin time ratio (PR) obtained from the patient’s PT value in seconds divided by the Mean Normal Prothrombin Time (MNPT), raised to the exponent power of ISI of the used PT reagent (*31*, *32*). The MNPT represents the geometric mean of PT values generated from a minimum of 20 healthy volunteers. The equation to obtain INR is as follows:

INR = (PR)^ISI^ or INR = [PT(s) / MNTP(s)]^ISI^,

where PT(s) is the patient’s PT value in seconds, PR is the prothrombin time ratio, MNPT is the mean normal prothrombin time and ISI is the international sensitivity index.

Although thromboplastins with ISI values up to 1.5 or even 1.7 are available on the market, it is recommended to use recombinant thromboplastins with lower ISI values, below 1.2, due to their greater sensitivities to factor deficiencies as well as precision improvement in INR determination (*29*-*33*). Regardless, an INR determined with different thromboplastins for an individual patient’s plasma sample is not always identical, as variations could also be generated mostly due to incorrect determination of MNPT or ISI value applied in laboratory (*31*).

Whenever it is possible ISI value specific for local thromboplastin/coagulometer combination should be used as it provides greater accuracy in INR over the use of a generic ISI (an ISI determined for a thromboplastin that is not coagulometer-specific but rather for a group of coagulometers). When ISI value for a specific thromboplastin/coagulometer combination is not available it is recommended to determine it locally (*29*, *30*, *32*). Briefly, this could be achieved by using a set of lyophilized plasma samples certified in terms of PT obtained by use of IRP and manual technique. Plasma should be tested by specific coagulometer/reagent combination and obtained PT values plotted against the certified values. The slope of the best fit orthogonal regression line can be used to calculate the local ISI according to equation: ISI (local) = SLOPE x ISI(IRP).

Determination of specific INR value without knowledge of ISI or MNPT values is possible by the direct INR method if lyophilized plasmas with assigned INR values are available. The calibration plasma is tested by specific coagulometer/reagent combination and coagulation times are plotted against the certified INR value. This method also increases the accuracy of INR determination and achieves better comparability of results between laboratories. For further details, readers are referred to references 29, 30 and 32.

**Recommendations**

1. Recombinant thromboplastins with ISI values below 1.2 should be used for PT determination.

2. ISI value for a specific combination of thromboplastin/coagulometer should be used whenever it is possible. If specific ISI is not available it should be determined locally.

### Activated partial thromboplastin time

The aPTT is a screening assay used for assessing deficiencies of FVIII, FIX, FXI and FXII or presence of their inhibitors. Each aPTT reagent contains a contact activator (*e.g.* silica, kaolin, ellagic acid or a combination of activators) and phospholipids of different origin, but as does not contain tissue factor (TF) it is called “partial thromboplastin”. Factors that are present in plasma samples are “activated” after the addition of aPTT reagent and calcium chloride as a separate reagent, at 37 °C (*29*, *34*).

Activated partial thromboplastin time is determined in seconds and results are highly dependent on reagent and instrument in use. Due to composition variability *i.e.* phospholipids and activators, individual aPTT reagents differ considerably in their factor, UFH and lupus anticoagulant (LA) sensitivities. For performing aPTT as a screening test, it is recommended to use aPTT reagent sensitive to factor deficiency and UFH therapy, but at the same time it does not have to be sensitive to LA (*4*, *29*, *34*).

However, monitoring of UFH therapy could be performed by aPTT, but it is quite difficult to standardize between different laboratories (*35*). To achieve this, general recommendation for laboratories is to establish therapeutic interval for UFH therapy monitoring according to anti-Xa activity corresponding to a range of 0.3 to 0.7 anti-Xa kIU/L. In brief, blood is collected from patients being treated with continuous intravenous UFH, at least 4-6 hours after a heparin bolus but also less than 24 hours after the first dose of warfarin. Blood samples should be processed according to standard procedure, preferably centrifuged twice and the plasma should be frozen at - 35 °C or lower until testing is performed. Plasma is thawed at 37 °C for 10 minutes and aPTT and heparin are measured by an anti-Xa test. The relationship between aPTT plotted on the Y-axis and heparin level plotted on the X-axis, is obtained by using linear regression analysis (*35*-*37*).

In contrast to UFH, low molecular weight heparin (LMWH) that has largely replaced the use of UFH in most clinical situations, does not require routine laboratory monitoring. However, in several clinical conditions and/or patient populations LMWH therapy monitoring is required, such as in patients with renal failure, obese patients, children and pregnant women or in case of any patient in whom the expected anticoagulant effect is not achieved. As LMWHs have predominantly anti-Xa activity and do not exhibit an adequate impact on aPTT, aPTT should not be used for LMWH monitoring. For estimating LMWH therapeutic response anti-Xa assay should be used exclusively (*38*).

**Recommendation**

1. To performing aPTT as a screening test, it is recommended to use aPTT reagents sensitive to factor deficiency and UFH therapy, but at the same time it does not have to be sensitive to LA.

2. aPTT assay is an appropriate assay for UFH therapy monitoring.

3. If aPTT is used for UFH therapy monitoring, general recommendation for laboratories is to test the sensitivity of aPTT reagents in order to establish appropriate therapeutic interval.

4. aPTT should not be used for LMWH monitoring. For estimating LMWH therapeutic response anti-Xa assay should be used exclusively.

### Thrombin time

The TT is a screening coagulation assay used for assessing deficiencies or qualitative fibrinogen disorders, or the presence of thrombin (activated FII, FIIa) inhibitor (*39*, *40*). It evaluates the ability of fibrinogen to be converted into fibrin after addition of bovine or human thrombin in excess to PPP. The TT is measured in seconds. Test is sensitive to anticoagulation therapy by thrombin inhibitors that may be present in plasma (*e.g.* heparin and dabigatran) and could detect accidental heparin contamination from catheters even at very low concentrations (> 0.05 anti-Xa kIU/L). However, due to high sensitivity to thrombin inhibitors (heparin, dabigatran), this assay is not adequate for monitoring anticoagulant therapy with heparin or dabigatran, and is not standardized for this purpose (*40*).

**Recommendation**

Thrombin time should not be used for monitoring anticoagulant therapy with heparin or thrombin inhibitors, as it is too sensitive and not standardized for this purpose.

### Fibrinogen

In most occasions, fibrinogen assay usually serves along with PT and aPTT testing as part of a general haemostatic screen (*41*, *42*). Several types of assays for measuring fibrinogen levels in plasma are available, but the most widely used assay measures functional fibrinogen activity based on the Clauss method (*42*). The principle of the Clauss method is that a high concentration of thrombin reagent is added to diluted plasma sample and clotting time is determined in seconds. As it is expressed in g/L, the test requires reference calibration plasma with known levels of fibrinogen calibrated against an International standard. Assigned and obtained values are plotted in order to generate a specific calibration curve covering a broad range of fibrinogen concentrations (*42*, *43*). Although effect of the reagent and coagulometer combination has a minimal effect on the Clauss assay, some investigations showed that differences could be obtained in certain subgroups of patients (*e.g.* disseminated intravascular coagulation (DIC), thrombolytic therapy) due to thrombin concentration and buffer in the reagent (*42*). Fibrinogen functional assay is generally considered as the most reliable method for widespread use in laboratories (*42*).

Immunoassays such as enzyme linked immunosorbent assay (ELISA) and immunonephelometric assays measure concentration of fibrinogen antigen, rather than its functional activity (*5*). These assays are mainly used in differential diagnosis of dysfibrinogenemia that is based on the difference in results obtained by measuring functional activity and concentration of fibrinogen antigen. In practice, such tests are only applied in highly-specialized laboratories (*42*).

Derived fibrinogen method allows an indirect fibrinogen level estimation from the PT clotting curve on automated optical coagulometers. The PT is determined by optical density change for a range of plasma dilutions with known fibrinogen levels and the optical change for each different fibrinogen level is plotted as a calibration curve. However, literature data have shown great controversy regarding its suitability for clinical use (*44*, *45*). Thus, although being a simple and inexpensive method, the test can give misleading results in particular disorders and clinical situations and is not recommended for routine laboratory use (*42*).

**Recommendations**

1. Fibrinogen functional assay is the most reliable method for routine use in clinical laboratory.

2. Fibrinogen immunoassays that measure concentration of fibrinogen rather than its functional activity are recommended in differential diagnosis of dysfibrinogenemia.

3. Derived fibrinogen method is not recommended routine laboratory use.

### D-dimer

For D-dimer measurement, different qualitative (positive or negative), semi-quantitative and quantitative methods, such as ELISA or latex immunoassay (LIA) which use a monoclonal antibody specific to various D-dimer epitopes are available (*46*, *47*). The main problem related to D-dimer testing is that various D-dimer assays are not standardized due to the variability in methodological differences, including the use of different monoclonal antibodies and calibrators for testing (*46*-*49*). As to date no standard reference preparation (*i.e.* international standard) is available, measurement units are still not standardized meaning that results, reference intervals and clinical cut-off values cannot be extrapolated between methods. Thus, D-dimer results must be carefully interpreted based on the assay used (*48*-*50*). On the test report along with the D-dimer results, it is necessary to point out the measurement method (*e.g.* measurement method enzyme-linked fluorescent assay, ELFA).

**Recommendations**

1. D-dimer results, reference intervals and cut-off values should be interpreted depending on method that laboratory use.

2. D-dimer measurement method should be pointed out on the test report.

## Postanalytical phase in coagulation testing

### Reference intervals, cut-off values and harmonisation of result reporting

Test results of most coagulation assays as well as reference intervals are strongly influenced by the combination of the reagent and instrument in use. Thus, if patients should use more than one laboratory for coagulation testing a lack of result comparability could exist.

According to literature, general recommendation for each laboratory is to determine own reference intervals for local population. However, as this is difficult to apply in daily practice most laboratories tend to use reference intervals recommended by the manufacturer or those available from the literature data (*25*, *51*-*53*). Determination of own reference intervals in practice is mainly applied when introducing a new method and literature data are not available. If reference intervals are taken from the manufacturer or from the literature, data on the population and the method of determination (applicability to the local population) should be checked and such reference intervals should be verified. To verify whether proposed values are appropriate for a local population, a sample consisting of a practical minimum, from 20 to 40 healthy subjects evenly spread through the published reference interval, depending on the required accuracy should be used. If 95% of the results fall within the published reference interval, it can be accepted for use. However, full statistical validity may require 120 subjects or even more (*25*, *53*). For some analytes such as D-dimers, it is more appropriate to establish cut-off values based on clinical utility considerations (*25*, *48*).

The use of age-adjusted reference intervals is critical for ensuring proper management of children with thrombosis or bleeding disorders, but published age-appropriate reference intervals are not available for the majority of reagents currently used in practice. The general recommendation of the SSC of the ISTH for each laboratory reporting paediatric samples is to define their own age-adjusted reference intervals for analyser and reagent in use (*54*). However, laboratories that are not able to determine their own paediatric reference intervals should adhere to and verify published age-adjusted reference intervals if these are available.

**Recommendations**

1. If reference intervals are adopted from the literature or manufacturer, it is recommended to verify if such intervals are appropriate for a local population.

2. The use of age-adjusted reference intervals is critical for ensuring proper management of children with thrombosis or bleeding disorders.

### Reporting of coagulation screening assays and D-dimer results

#### Reporting of PT/INR results

The prerequisite for correct reporting of PT results is adequate information on patient`s test request (suspected diagnosis and/or patient`s therapy). Understanding the relevance of different result reporting in individual clinical conditions could directly result in a better management of patients.

The PT test is measured in seconds whereas blood coagulability is typically expressed as “percentage activity of the normal value”. In an adult person not receiving VKAs, the “normal” PT value is > 70%. A value lower than 70% indicates that the clotting time is longer than normal and suggests blood hypocoagulability (*29*, *51*).

Considering VKA therapy monitoring, the INR system for reporting PT results across different laboratories should be used exclusively, whereas in all other clinical indications INR should not be reported (*30*). Reporting results as PT-ratio cannot be used for reliable dose-adjustment for patients on VKA therapy as this could only be applicable to a particular brand of thromboplastin. However, some authors consider that in certain conditions such as chronic liver disease or DIC reporting results as PT-ratio is informative (*33*, *55*).

In general, INR values between 2.0 and 3.0 are the proposed therapeutic interval for the majority of clinical indications, although for mechanical heart valves the target INR range might be slightly higher (2.0-3.5) (*30*, *33*). INR values above the recommended therapeutic interval indicates increased bleeding risk, whereas values below the therapeutic interval are associated with increased risk of thrombosis. It is important to note that INR value should always be reported as a numerical value up to a certain measurement limit, which is variable depending on the calibration curve obtained for a specific reagent lot. It is not perfect in terms of results comparability between laboratories but it reduces the variation and provides clinically useful results (*29*, *30*).

**Recommendations**

1. PT results should be reported as percentage if patients are not on VKA therapy.

2. INR should not be reported for patients not receiving VKA.

3. The INR system for reporting PT results should be used exclusively for VKA therapy monitoring.

#### Reporting of aPTT results

The aPTT test is both measured and reported in seconds. Correct interpretation of aPTT test results requires understanding of the clinical context in which the test is ordered as well as the test’s limitations (*29*, *34*). As aPTT reagents differ considerably in their sensitivity to coagulation factor deficiencies, UFH and LA (*29*, *34*-*37*), reporting aPTT results in seconds only, can lead to inappropriate comparability of results between different laboratories since both reference intervals and test result vary between different reagents and coagulometers. Therefore, an aPTT ratio should always be reported together with the result expressed in seconds.

The aPTT ratio is calculated as the ratio of patient’s aPTT divided by the mean value of the reference interval for the particular system (reagent/coagulometer) used (*51*). Considering aPTT reference interval in seconds, it could be taken from manufacturer or from the literature but applicability to the local population and verification should be performed. Reference interval expressed as aPTT ratio generally is between 0.8-1.2, independently of the system in use.

**Recommendation**

The aPTT ratio should always be reported together with the result expressed in seconds.

#### Reporting of TT results

The TT test has little diagnostic value by itself and should be interpreted within the context of the first line coagulation assays, including PT, aPTT and fibrinogen. The TT test is measured and reported in seconds. Reference intervals are still not harmonised and are highly dependent on the combination of reagent in use (*i.e.* thrombin concentration in the TT reagent) and coagulometer (*39*, *40*). As for aPTT, reference interval could be adopted from manufacturer or from the literature but verification should be performed.

**Recommendation**

The TT test should be reported in seconds.

#### Fibrinogen

Results of fibrinogen activity test are expressed as g/L. In healthy adults, the reference interval for fibrinogen is generally between 1.8-4.0 g/L but it could be different for diverse commercial fibrinogen tests (*42*). Recent survey among Croatian laboratories showed that laboratories use reference interval according to document on harmonisation published by CCMB in 2005 but as it is based on the results obtained from 3.8% citrate tubes this certainly should be revised (*6*, *33*). Reference interval for fibrinogen could be adopted from manufacturer or from the literature but verification should be performed.

**Recommendation**

Results of fibrinogen activity test should be expressed as g/L.

#### D-dimer

Different D-dimer methods employ monoclonal antibodies with different specificities, different measurement units and cut-off values. Therefore, it is not possible to compare results obtained by different methods (*46*-*50*). In general, D-dimer results in fibrinogen-equivalent units (FEU) are roughly two-fold higher than D-dimer units (DDU) results (*i.e.* 1.0 mg/L FEU is approximately the equivalent of 0.5 mg/L DDU). The consensus regarding the preferable reporting unit is still not reached. Moreover, another problem encountered in daily practice is that package inserts of particular approved assay kits do not provide information about the type of unit (DDU or FEU) used in the assay. Thus, use of such assay kits could not be recommended. Furthermore, there is variability in D-dimer result reporting related to measurement units, such as mg/L or µg/L, currently in use, that could have a confusing effect on clinicians (*46*, *48*). Since results of different methods for D-dimer testing cannot be compared, laboratories should declare on the test report their method in use along with an appropriate unit related to DDU or FEU. Considering measurement units, results should be expressed as mg/L DDU or FEU.

General recommendation for each laboratory is to determine its own cut-off values for particular D-dimer method in current use (*48*). However, since it is quite difficult to achieve this requirement for almost all laboratories, cut-off values are mostly relied on manufacturer´s declared values. It is recommended that such cut-off values should be clinically validated. It should be stressed that cut-off values for D-dimers reflect high negative predictive value of testing and the main value of test is the absence of elevated values.

**Recommendation**

1. D-dimer results should be expressed in mg/L DDU or FEU.

2. D-dimer measurement method in use should be declared on the test report along with the test result.

3. Assay kits that do not provide information about the type of unit used (DDU or FEU) in the assay should not be used.

### Interpretative comments

Recent survey among diagnostic laboratories in Croatia showed that only small number of laboratories interprets the results of the coagulation test report or recommendations for further testing (*6*). Nowadays, as an integral part of laboratory service, laboratories are increasingly encouraged to provide interpretative and/or informative comments related to interferences and findings discovered by the laboratory for a particular patient´s sample (*56*). The interpretative and/or informative comments attached to the result report may help the clinician to appropriately use the laboratory information. Thus, in order to prevent or reduce errors and improve patient outcomes, the practice of adding appropriate interpretative comments, related to the both preanalytical and analytical phases of testing, as well as those related to the extension of the original clinical request, are strongly recommended and should be an integral part of the test report whenever it is justified. Harmonization of interpretative comments exceeds the scope of these guidelines, yet wherever it was possible, the comments were given.

**Recommendation**

Interpretative comments, related to the both, preanalytical and analytical phases of testing, as well as those related to the extension of the original clinical request, are strongly recommended and should be an integral part of the test report whenever it is justified.

### Critical values reporting

Critical results are the values that represent a pathophysiological condition at such variance compared to the normal one (expected values) or previously obtained values, as to be life threatening and for which some corrective action should be taken promptly. Considering haemostasis these could be significant for an imminent risk of severe bleeding or thrombosis (*57*).

In general, critical values for PT, aPTT and fibrinogen are variable worldwide and are still not standardized. In clinical practice, INR value > 5.0 is considered clinically relevant as it requires urgent intervention in order to reduce the warfarin anticoagulant effect (*e.g.* withholding warfarin doses) and also mitigate bleeding risk (*e.g.* administration of vitamin K, fresh frozen plasma or prothrombin complex) (*58*, *59*). When setting critical value limits for the aPTT, laboratories need to consider the aPTT results specific for their reagent and instrument combination according to previously described procedure (*59*). In general, therapeutic interval for the aPTT ratio should be 1.5 to 2.5 times greater than the upper limit of the reference interval and any greater ratio could indicate increased bleeding risk and might merit urgent action (*e.g.* readjustment of heparin dosing) (*57*). Thus, it should be discussed with a clinician, as so far, there is no consensus regarding appropriate critical value. Furthermore, fibrinogen values lower than 0.8 g/L would also indicate an elevated bleeding risk and should be immediately reported to a clinician (*57*, *59*). However, in the absence of widely accepted guidelines, laboratories are encouraged to define locally clinical relevant critical limits and/or expand the existing list of critical values in conjunction with local clinical opinion (*57*). However, rules of critical values reporting may differ for a first time sample *vs.* repeated patient samples. The first critical result should be reported immediately to the physician, and depending on the agreement with the clinical staff, reporting of the each following critical results for the same patient should be managed. Therefore, it is important to note that critical value reporting policy in each institution should be result of joint work between laboratory and clinical personnel (*60*).

**Recommendations**

1. Coagulation laboratories are encouraged to define locally clinical relevant critical limits and/or expand the existing list of critical values in conjunction with local clinical opinion.

2. The first critical result should be reported immediately to the physician, and depending on the agreement with the clinical staff, reporting of the each following critical results for the same patient should be managed.

3. INR value > 5.0 is considered clinically relevant as it requires urgent intervention in order to reduce the warfarin anticoagulant effect.

4. Therapeutic interval for the aPTT ratio should be 1.5 to 2.5 times greater than the upper limit of the reference interval and any greater ratio should be dis-cussed with clinicians as it could indi-cate increased bleeding risk.

5. Fibrinogen values lower than 0.8 g/L could be indicative for an elevated bleeding risk and should be immediately reported to a clinician.

## Summary

Recent survey in Republic of Croatia showed substantial variability in practice and policies among laboratories performing coagulation testing (*5*). This is not surprising as laboratories operate at different healthcare levels and under different professional societies. Thus, the objective of this review was to provide the basic recommendations in preanalytical, analytical and postanalytical phases of testing for most commonly performed coagulation assays. However, differences in technological solutions among the laboratories with a small and a large volume of samples could exist. Coagulation testing is also increasingly integrated into total laboratory automation systems in order to improve quality, efficiency and patient care, while reducing costs, but the principle of analyses remains the same. Independently of the number of tests or integration, haemostasis testing requires specific skills (*61*). Appropriate use of obtained information is of vital importance as well as recognition of the potential sources of error through the total laboratory process. Thus, we hope that these recommendations would be helpful in every day practice for laboratories performing routine haemostasis assays. As gathered information are based on previously published guidelines, expert committees’ reports or expert consensus opinion, the strength of evidence is not so pronounced. Nevertheless, we consider these recommendations as an important step towards standardization of procedures and generate mutually acceptable data among Croatian laboratories (Appendix 1).

## Appendix 1 Basic recommendations for procedures in preanalytical, analytical and postanalytical phases of prothrombin time, activated partial thromboplastin time, fibrinogen and D-dimer testing

**Table:**

**Phase of coagulation testing**		**Recommendation**	**Reference(s)**
**Preanalytical phase in coagulation testing**			
**Test request**			
	Information on suspected or established diagnosis as well as on anticoagulant therapy should be an integral part of the coagulation test request.		10
**Patient preparation and identification**			
	Preparation of patient prior to venipuncture and appropriate identification of patient during venipuncture should be in compliance to National recommendations for venous blood sampling.		10
**Sample tubes and anticoagulant**			
	1. Venous blood specimens for coagulation testing should be collected into glass or plastic test tubes, both containing non-activating surfaces.		11,13
	2. Test tubes should contain buffered trisodium citrate as anticoagulant, preferably at concentration of 105-109 mmol/L, also referred as 3.2% trisodium citrate.		11
	3. The proportion of blood to anticoagulant volume should always be 9:1.		11
	4. Samples with +/- 10% of the fill line, corresponding to 90-110% fill volume could be accepted for analysis.		11,12-14
**Blood sample collection**			
	1. Collection of blood samples for coagulation testing should be in compliance to National recommendations for venous blood sampling.		10
	2. Prior to collection of specimen for coagulation testing through an intravenous catheter, it is recommended to flush the central catheter line with saline and to discard first 5 mL of blood or 6-times of the line volume or if collected from a capped off peripheral venous catheter, twice of the catheter and extension set dead space volume should be discarded.		8,10,11,15
**Order of test tubes draw**			
	1. The coagulation tube has to be collected preferably before any other tube with additive (by clot activator or anticoagulant).		11,13,14
	2. There is no need to first draw a discard tube prior to collecting specimens for routine coagulation tests and D-dimers. The exception of this rule includes a procedure when a winged blood collection set should be used, as the air in the tubing leads to the under filling of the test tube. A discard tube in this case should be a non-additive.		10,11,14
**Specimen rejection**			
	Each laboratory should have defined criteria for rejecting unsuitable specimens for coagulation testing.		8,11,16
**Sample processing**			
	1. Plasma samples for determination of screening assays, aPTT, TT, fibrinogen as well as D-dimers, should be prepared by centrifugation of primary collection tube at ambient temperature (18-25 °C), at 1500xg for 15 minutes.		11,13,17
	2. All plasma samples that should be frozen until analysis, should be double centrifugated prior to freezing to obtain platelet poor plasma (PPP), containing < 10 x10^9^/L platelets.		16,17
**Samples with high haematocrit values**			
	1. The final concentration of the citrate in the test tube should be adjusted in order to maintain the appropriate blood to anticoagulant ratio at 9:1 in samples with haematocrit values above 0.55 L/L.		11,18
	2. On the test report, it should be always indicated that citrate volume in the test tube was adjusted due to high haematocrit values: “Coagulation assays are performed in samples with adjusted volume of anticoagulant (citrate) due to high haematocrit value (Hct = xx L/L).”, where xx represents the exact amount of haematocrit.		11,13,18
**Haemolysis, hyperbilirubinemia and lipemia**			
	1. For coagulation screening assays laboratories should evaluate acceptable concentration of interfering substances for their own system (reagent/coagulometer).		19,20
	2. If intravascular or *in vivo* haemolysis is suspected, along with the results appropriate notation on the test report (intravascular haemolysis) should be given.		11,19,20
	3. Use of mechanical and/or electromechanical clot detection methods is recommended whenever it is possible for samples that contain substances interfering with light transmission.		11
**Sample storage until analysis**			
	1. Specimens for all haemostasis assays should be kept capped (in an unopened tube) at ambient temperature (18-25 °C) until analysis.		11,12,20
	2. Testing for all coagulation assays should be performed within 4 hours after blood collection. The exception of this rule could be applied to PT and D-dimer testing, for which specimens can be stored at ambient temperature for up to 24 hours after blood collection, either uncentrifuged or centrifuged with plasma remaining on the top of the cellular component in an unopened tube.		11,20,21
	3. Laboratories that accept specimens for PT and D-dimer testing stored within 24 hours of blood collection should confirm sample stability with their own system.		21
	4. If aPTT testing is requested for UFH monitoring, specimens should be centrifuged and plasma should be removed from cells within 1 hour of collection.		11,16,21
	5. If testing for any haemostasis assay is not possible within the allowed time, plasma should be removed from the cells after centrifugation and immediately frozen at – 20 °C or below for short-term storage (up to two weeks) or at – 70 °C for up to six months.		16,20
**Transport of specimens to distant laboratories**			
	1. If coagulation specimens should be sent to distant laboratories for analysis, samples should arrive in the testing laboratory in real time to allow analysis within appropriate timeframes specified for each assay. If it is not possible to transport specimens within appropriate timeframes for analysis, blood samples should be processed as it is described in previous section as recommendation 5.		16,20
	2. Each vial with specimen that should be sent at distant laboratories for analysis, should be labelled with the patient’s full name, date of birth, identification number (insurance number or unique identification number in laboratory). In addition, sample type (*e.g.* citrate plasma), date and time of sampling should be also provided.		16,20
	3. Frozen plasma specimens should be transported preferable on a dry ice, and if not possible on sufficient amount of regular ice in a Styrofoam container in order to keep the samples solidly frozen until they arrive at the laboratory.		5,8,11
**Thawing of frozen plasma samples**			
	1. Thawing of frozen plasma samples should be performed rapidly, for 10 minutes, at 37 °C in an incubator, dry thermo block or water bath.		5,16,20,23
	2. To ensure sample integrity prior to testing, thawed sample should be thoroughly mixed by the gentle inversion of sample for 180° and return to starting position for 6-times.		23
**Analytical phase in coagulation testing**			
**Quality control**			
	1. Internal quality control (IQC) plasma should be analysed always following opening or/and reconstitution of new reagent, calibration, preventive coagulometer maintenance and repair.		27
	2. For quantitative coagulation tests, as a minimum two levels of control material including both normal and pathological control plasma should be run every eight hours of continuous operation. Different IQC schedule could be allowable if laboratory has analysed its own working process and performed the risk analysis prior to use.		27,29
	3. External quality assessment should be essential part of the total quality management in coagulation laboratory.		27,29
**Coagulation assays**			
	For implementation of coagulometers into daily practice, evaluation and validation of procedures should be performed in compliance to previously published recommendations.		26
**Prothrombin time**			
	1. Recombinant thromboplastins with ISI values below 1.2 should be used for PT determination.		29-33
	2. ISI value for a specific combination of thromboplastin/coagulometer should be used whenever it is possible. If specific ISI is not available it should be determined locally.		29,30,32
**Activated partial thromboplastin time**			
	1. To perform aPTT as a screening test, it is recommended to use aPTT reagents sensitive to factor deficiency and UFH therapy, but at the same time they do not have to be sensitive to LA.		4,29,34
	2. aPTT assay is an appropriate assay for UFH therapy monitoring.		35
	3. If aPTT is used for UFH therapy monitoring, general recommendation for laboratories is to test the sensitivity of aPTT reagents in order to establish appropriate therapeutic interval.		35-37
	4. aPTT should not be used for LMWH monitoring. For estimating LMWH therapeutic response anti-Xa assay should be used exclusively.		38
**Thrombin time**			
	Thrombin time should not be used for monitoring anticoagulant therapy with haeparin or thrombin inhibitors as it is too sensitive and not standardized for this purpose.		39,40
**Fibrinogen**			
	1. Fibrinogen functional assay is the most reliable method for routine use in clinical laboratory.		42
	2. Fibrinogen immunoassays that measure concentration of fibrinogen rather than its functional activity are recommended in differential diagnosis of dysfibrinogenemia.		39,42
	3. Derived fibrinogen assay is not recommended for routine laboratory use.		42,44,45
**D-dimer**			
	1. D-dimer results, reference intervals and cut-off values should be interpreted depending on method that laboratory use.		47,48
	2. D-dimer measurement method should be pointed out on the test report along with the test results.		48-50
**Postanalytical phase in coagulation testing**			
**Reference intervals and cut-off values and harmonisation of result reporting**			
	2. If reference intervals are adopted from the literature or manufacturer, it is recommended to verify if such intervals are appropriate for a local population.		25,53
	3. The use of age-adjusted reference intervals is critical for ensuring proper management of children with thrombosis or bleeding disorders.		54
**Reporting of coagulation screening assays and D-dimer results**			
**Reporting of PT/INR results**			
	1. PT results should be reported as percentage if patients are not on VKA therapy.		29,30,51
	2. INR should not be reported for patients not receiving VKA.		30
	3. The INR system for reporting PT results should be used exclusively for VKA therapy monitoring.		29,30
**Reporting of aPTT results**			
	The aPTT ratio should always be reported together with the result expressed in seconds.		29,35-37
**Reporting of TT results**			
	The TT test should be reported in seconds.		39,40
**Reporting of fibrinogen results**			
	Fibrinogen functional assay should be reported in g/L.		42
**Reporting of D-dimer results**			
	1. D-dimer results should be expressed as mg/L DDU or FEU.		46-48
	2. D-dimer measurement method in use should be declared on the test report along with the test result.		46-48
	3. Assay kits that do not provide information about the type of unit used (DDU or FEU) in the assay should not be used.		46-48
**Interpretative comments**			
	Interpretative comments, related to the both, preanalytical and analytical phases of testing, as well as those related to the extension of the original clinical request, are strongly recommended and should be an integral part of the test report whenever it is justified.		56
**Critical values reporting**			
	1. Coagulation laboratories are encouraged to define locally clinical relevant critical limits and/or expand the existing list of critical values in conjunction with local clinical opinion.		57,59
	2. The first critical result should be reported immediately to the physician, and depending on the agreement with the clinical staff, reporting of the each following critical results for the same patient should be managed.		57,59
	3. INR value > 5.0 is considered clinically relevant as it requires urgent intervention in order to reduce the warfarin anticoagulant effect.		58
	4. For UFH monitoring, any aPTT ratio greater than 2.5 times of the upper limit of the reference interval should be discussed with clinicians as it could indicate increased bleeding risk.		57
	5. Fibrinogen values lower than 0.8 g/L could indicate an elevated bleeding risk and should be immediately reported to a clinician.		57,59
PT - prothrombin time. aPTT - activated partial thromboplastin time. TT - thrombin time. ISI- International Sensitivity Index. INR - International Normalized Ratio. LMWH - low molecular weight heparin. UFH - unfractionated heparin. VKA - vitamin K antagonist. DDU - D-dimer units. FEU - fibrinogen equivalent units. LA - lupus anticoagulant.			
